# Deep learning for irregularly and regularly missing data reconstruction

**DOI:** 10.1038/s41598-020-59801-x

**Published:** 2020-02-24

**Authors:** Xintao Chai, Hanming Gu, Feng Li, Hongyou Duan, Xiaobo Hu, Kai Lin

**Affiliations:** 1 China University of Geosciences (Wuhan), Institute of Geophysics and Geomatics, DeepResearch Group, Center for Wave Propagation and Imaging, Wuhan, Hubei China; 2 Sinopec Henan Oilfield Branch Company, Nanyang, Henan China; 3 Henan Oilfield Exploration and Development Research Institute, Zhengzhou, Henan China

**Keywords:** Geophysics, Seismology

## Abstract

Deep learning (DL) is a powerful tool for mining features from data, which can theoretically avoid assumptions (e.g., linear events) constraining conventional interpolation methods. Motivated by this and inspired by image-to-image translation, we applied DL to irregularly and regularly missing data reconstruction with the aim of transforming incomplete data into corresponding complete data. To accomplish this, we established a model architecture with randomly sampled data as input and corresponding complete data as output, which was based on an encoder-decoder-style U-Net convolutional neural network. We carefully prepared the training data using synthetic and field seismic data. We used a mean-squared-error loss function and an Adam optimizer to train the network. We displayed the feature maps for a randomly sampled data set going through the trained model with the aim of explaining how the missing data are reconstructed. We benchmarked the method on several typical datasets for irregularly missing data reconstruction, which achieved better performances compared with a peer-reviewed Fourier transform interpolation method, verifying the effectiveness, superiority, and generalization capability of our approach. Because regularly missing is a special case of irregularly missing, we successfully applied the model to regularly missing data reconstruction, although it was trained with irregularly sampled data only.

## Introduction

Deep learning (DL)^1^ is a branch of machine learning (ML) that addresses the question of how to build computers that intelligently improve through experience^2^. Recently, DL or ML, in general, enjoyed an explosive growth and showed great promise in various areas, e.g., biology^3,4^, image reconstruction^5,6^, and solid earth geoscience^7^. DL is powerful for mining features or relationships from data, which is invaluable in the context of big data, as it extracts high-level information from huge volumes of data. Please refer to Goodfellow *et al*.^8^ for a good textbook of DL. One of the most popular DL technologies is the convolutional neural network (CNN), which is at the core of most state-of-the-art DL solutions for numerous tasks^9^. In recent years, deep CNNs have had stunning successes, surpassing human accuracy for hard problems such as visual recognition^6^.

In exploration seismology, DL or ML has been widely used in fault detection^10^, structural interpretation^11^, inversion^12^, and data interpolation^13–15^, to name a few. A more tremendous trend of developments has recently come about through the use of DL not for image analysis but for image transformation. In these cases, CNNs are trained to transform one type of image into another. Many geophysical problems can be posed as transforming an input profile into a corresponding output profile (e.g., denoising: transforming noisy data to noise-free data). Inspired by Isola *et al*.^16^, where DL is investigated as a general-purpose solution to image-to-image translation problems, we apply DL to missing data reconstruction with the aim of transforming an input incomplete data set into a corresponding complete data set, which is an important ongoing research topic in exploration seismology.

Physical (e.g., the presence of obstacles, no-permit areas, and hardware problems with geophones/hydrophones/air-guns) and economic constraints lead seismic data to be incomplete or sparsely sampled during data acquisition^17^. However, many important techniques cannot adequately handle irregular sampling and rely on uniformly and densely sampled, unaliased input data (e.g., 2D/3D surface-related multiple elimination, amplitude-variation-with-offset analysis, and reservoir characterization). The performance of multichannel data processing depends heavily on the spatial sampling intervals. Too large an interval leads to aliasing, adversely resulting in poor resolution. Therefore, the missing data should be reconstructed^18^.

The missing data problem can be classified into two categories: regularly missing and irregularly missing. Regularly missing means the data are equidistantly or periodically missing at a constant rate in uniform grids. Irregularly missing means the data are randomly missing on uniform grids^19^. Seismic data are often irregularly and sparsely sampled along the spatial coordinates, leading to suboptimal processing and imaging results. This work primarily concentrates on solving the irregularly missing data problem. Solving the regularly missing data reconstruction problem is an unexpected harvest.

Based on a variety of principles and assumptions, important advances have been made in seismic data reconstruction. Some of them addressed interpolating regularly sampled data^13–15,20^, while some of them attacked non-uniformly sampled interpolation^21^. There are techniques developed for both irregularly and regularly missing data reconstruction^22^. A complete and detailed discussion of previous publications is beyond the scope of this work. We only review some key viewpoints and literature closely related to the subject of this work. The methods based on classical signal-processing principles use specific properties of seismic data as a priori information for interpolation. Signal-processing reconstruction techniques via transforming the data to other domains and prediction-error filtering generally assume that the data are composed of a superposition of a few plane waves. The sparseness, band limitation, and low-rank assumptions also underlie some of these methods.

Naghizadeh and Innanen^23^ addressed seismic data interpolation using a fast-generalized Fourier transform (FGFT). They utilized the FGFT to identify the space-wavenumber evolution of spatial signals at each temporal frequency, and a least-squares fitting scheme to retrieve the optimal FGFT coefficients representative of the desired interpolated data. For randomly sampled data, they sought a sparse representation of FGFT coefficients to retrieve the missing pixels. To interpolate the regularly sampled data at a given frequency, they used a mask function derived from the FGFT coefficients of the low frequencies. This makes the FGFT interpolation method a good competitor, which is used for comparison in our work.

Of the multitudinous methods for seismic data interpolation, few take advantage of recent developments in DL or ML. Jia and Ma^13^ proposed a method for reconstructing seismic data from regularly under-sampled traces based on a classic ML method of support vector regression. Jia *et al*.^14^ proposed an intelligent interpolation method for regularly sampled data by Monte Carlo ML. Wang *et al*.^15^ proposed a DL-based approach for regularly sampled seismic data antialiasing interpolation. Based on CNNs, Wang *et al*.^15^ designed eight-layer residual networks (ResNets) with a better back-propagation property for interpolation, which extract feature maps of the training data in a non-linear way. For the methods of Jia and Ma^13^, Jia *et al*.^14^, and Wang *et al*.^15^, in the training process, to generate the input of the designed network, calculation of the initial pre-interpolation data using a bicubic method is required, which affects the final performance of their methods.

With the DL theory described in Goodfellow *et al*.^8^, we applied DL to both irregularly and regularly missing data reconstruction, where we defined intelligent data-to-data translation as the task of translating incomplete data into complete data (without pre-interpolation). DL allows trainable models composed of multiple layers to learn representations of data with multiple levels of abstraction^1^. DL is a representation-learning method with multiple levels of representation obtained by composing non-linear modules, in which each transforms the representation at one level into a representation at a higher, slightly more abstract level^8^. With the composition of enough such layers, and given sufficient training data, very complicated functions/relations can be learned. Moreover, as a motivation, DL can theoretically avoid some assumptions restricting conventional interpolation methods (e.g., assumptions of linear events, sparsity, band limitation, and low-rank).

The rest of the paper is organized as follows. In the “Methods” section, first, we briefly transcribe some basic DL theory; next, with the incomplete data as the model input and the corresponding complete data as the model output, we elaborate the established model architecture in detail, which is based on an U-Net convolutional network^4^; then, we provide detailed training analysis including the loss function definition, optimizer used, evaluation metrics, training data preparation, and parameter setup. In the “Results” section, to make the results more convincing and to validate the generalization capacity of the trained model, we test the model’s performance using several typical data sets (i.e., a synthetic training data set, a synthetic test data set, a physical modelling data set, the Mobil Viking graben line 12 data set, the F3 data set, a fault data set from the GeoFrame software, and a data set from the North Sea). The trained model is used to accomplish both irregularly and regularly missing data reconstruction. After discussing some practical aspects and extensions of this work, we summarize some concluding remarks.

## Methods

### Basic theory

For the sake of brevity, we refer the reader to Goodfellow *et al*.^8^ for a detailed description of the DL terminologies. DL is a computational tool to learn complex motifs from data. DL uses multiple processing layers to discover patterns and structures in the data. Each layer learns features from the data that subsequent layers build on. Non-linear parameterized processing layers are combined to progressively transform an input **X** into the desired output **Y**_ref_, typically attaining only approximations ${{\bf{Y}}}_{\mathrm{pre}}$. Deep artificial neural networks (ANNs) are black-box models whose operation is opaque and difficult to interpret. The adjective “deep” refers to the large number of stacked layers required for building a universal function approximator *f* as follows:

1 $${{\bf{Y}}}_{\mathrm{pre}}=f\left({\bf{X}};{\theta }\right),$$

where *θ* denotes the parameters (including but not limited to, weights **W** and biases **b** of the convolution kernels) in the CNNs.

We regard ANNs as bridges connecting the input **X** and the desired output **Y**_ref_. ANNs with a sufficient number of parameters can theoretically approximate any function. For fitting the desired mapping, the model needs to go through a training process, which begins with a random choice for *θ*. The training process can be considered as an optimization problem composed by finding a set of the internal parameters *θ* through the minimization of the discrepancy between ${{\bf{Y}}}_{\mathrm{pre}}$ and **Y**_ref_ (quantified by the loss function *ϕ*, which will be explained later) for all the samples fed into the model^12^.

The parameters *θ* are iteratively updated to minimize the loss using gradient descent and to improve the accuracy of the model prediction. Each layer of the model is differentiable, meaning that it is known how changes in *θ* cause changes in output values. The back-propagation (BP) algorithm^24^ uses the chain rule to efficiently compute all partial derivatives, or gradients, with just one forward pass through the model followed by a backward pass. The training process is accomplished if the loss function achieves an acceptable level. The optimizer for the loss function is an important requirement for training a model.

### Model architecture

**Figure 1:**
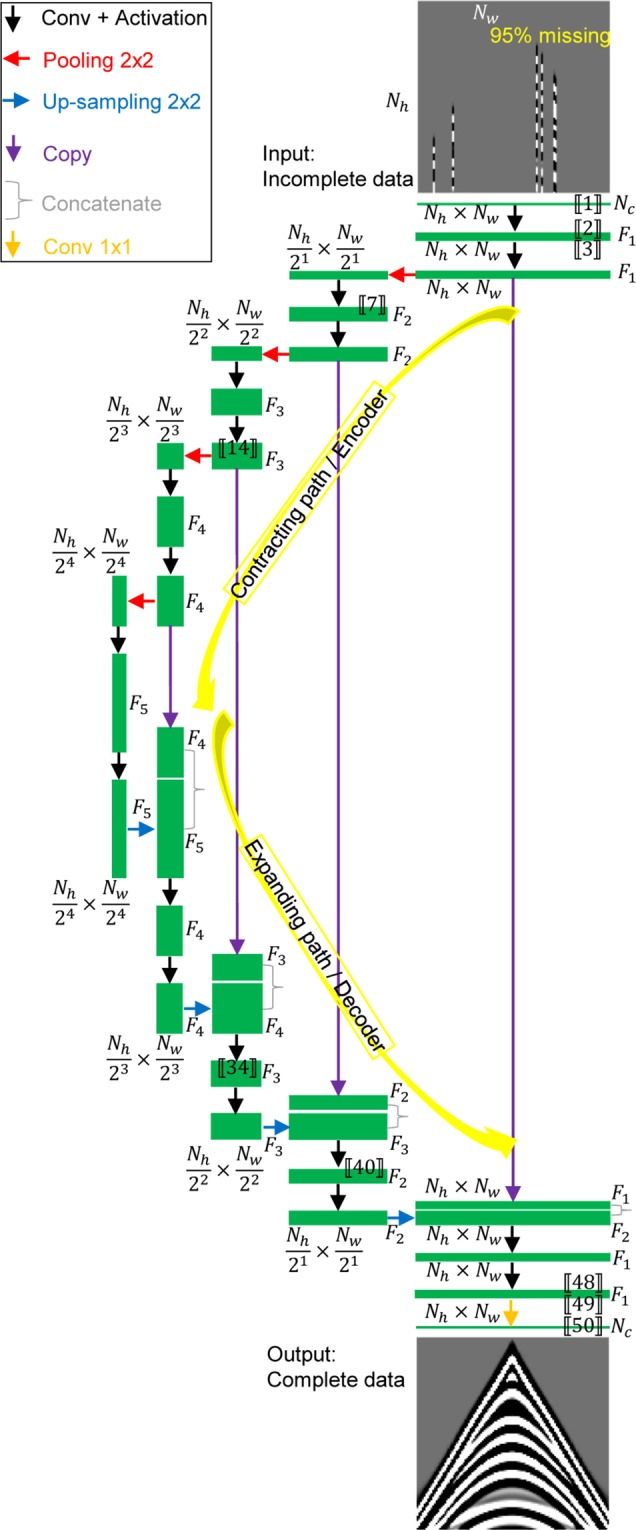
Network architecture at an example of $\frac{{N}_{h}}{{2}^{4}}\times \frac{{N}_{w}}{{2}^{4}}$ pixels in the lowest resolution. *N*_*h*_, *N*_*w*_, and *N*_*c*_ are the height, width, and the number of channels of the input data, respectively. Blocks show the calculated feature hierarchy. Each green box denotes multiple feature maps, and the number of feature maps (i.e., *F*_*i*,*i*∈[1, 5]_) is marked on the right of the box. The height-width-size of a feature map is given around the box. The boxes with the same height have the same number of feature maps. The boxes with the same width indicate the same height-width-size of feature maps. The arrows and the right curly brace denote different operations. Numbers in [[⋅]] are labelled according to Table 1, which are in line with those shown in Fig. 2.

There is no hard and fast rule for how many layers are needed to constitute ANNs, but most researchers agree that no less than three are required. Figure 1 shows a schematic of the established model architecture, which belongs to a specific family of neural network (NN) architectures known as U-Net, a generic DL solution for various tasks^4^. Specific to our mission, the model input data include one horizontal (spatial) dimension and one vertical (temporal) dimension. In seismic terminology, *N*_*h*_ and *N*_*w*_ denote the sampling points of the input data along the time and space axes, respectively. The number of channels *N*_*c*_ equals 1 for seismic data. A data sample with *N*_*c*_ channels is fed into the model on the top. The model input data **X** are randomly missing in accordance with a certain percentage (e.g., 40% → 95%) in the space direction. Please note that no pre-interpolation process is involved. The model output **Y**_ref_ (at the bottom) is the corresponding complete data.

**Figure 2:**
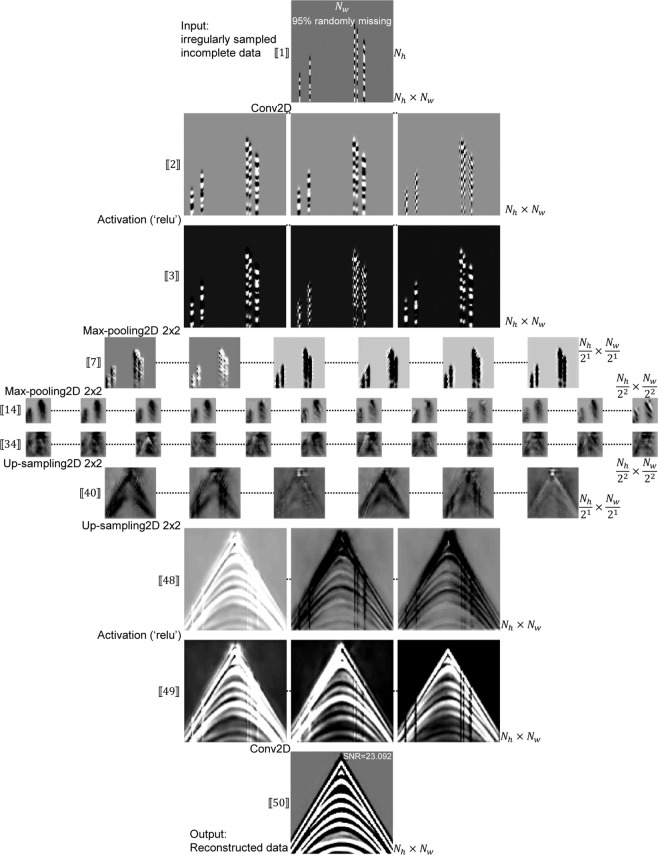
Feature maps for a randomly sampled data set going through the trained model. Numbers in [[⋅]] are labelled according to Table 1, which are consistent with those shown in Fig. 1. The symbol “...” implies the omitted feature maps.

The model architecture (Fig. 1) is an encoder-decoder-style NN solving the missing data reconstruction task end-to-end, which is logically composed of a contracting path (upper-side, interpreted as an encoder) and a more or less symmetric expanding path (lower-side, interpreted as a decoder). The encoder takes an incomplete data sample as input and gradually calculates feature maps at multiple scales and abstraction levels resulting in a multi-level, multi-resolution feature representation. Layers in the decoder successively synthesize the complete data starting at low-resolution feature maps (denoting large scale structures) up to high-resolution feature maps (representing fine scale structures)^4^. Please see Fig. 2 for a better understanding.

The contracting path/encoder follows a typical CNN, consisting of the repeated application of two padded convolutions (padding avoids the loss of border pixels in every convolution), each followed by an activation operation (black-arrow) and a pooling operation (red-arrow) with stride two halving the resolution of the resulting feature map. Convolutions directly following down-sampling steps double the number of feature maps^4^. Each step in the expansive path/decoder consists of a bed-of-nails up-sampling of the feature maps by a factor of two (blue-arrow) followed by a concatenation (right curly brace) with the copied encoder feature maps at the corresponding resolution (purple-arrow), and two convolutions, each followed by an activation. The feature maps from the contracting path are combined with the up-sampled output. Successive convolution layers then learn to assemble a more precise output based on this. Skip connections have been shown to help train deeper networks by preventing vanishing gradients^25^. At the final layer, a 1 × 1 convolution (green-arrow) is utilized to map multiple feature maps to the desired output **Y**_ref_.

A widely used non-linear activation function is the rectified linear unit (ReLU), which returns element-wise $\max ({\bf{x}},0)$ with **x** being an input tensor. The ReLU activation was adopted in the constructed model. For the pooling operation, we employed the max-pooling layer. Note that the number of output filters in the convolution (i.e., *F*_*i*,*i*∈[1, 5]_) increases (e.g., from 64 to 128, 256, 512, and 1024) as we go deep in the model. At each down-sampling step, we generally double the number of feature maps.

Table 1 provides a specified model summary. There are fifty layers (including 19 convolution layers). The trainable parameters focus on convolution layers. No trainable parameters exist in the input, ReLU activation, max-pooling, up-sampling, and concatenate layers. The trainable parameters in a convolution layer are computed by the following:

2 $$({K}_{h}\times {K}_{w}\times {F}_{i-1}+1)\times {F}_{i},$$

where *K*_*h*_ × *K*_*w*_ is the convolution kernel size, *F*_*i*−1_ and *F*_*i*_ denote the number of feature maps in the previous and current layers, respectively. +1 means a bias is added. Figure 2 shows the feature maps for a randomly missing data sample going through a trained model. Once trained, it is capable of filling in the gaps in corrupted data by going through encoding and decoding steps.

**Table 1:** Model summary for the model architecture shown in Fig. 1, where *N*_*c*_ = 1, *F*_1_ = 64, *F*_2_ = 128, *F*_3_ = 256, *F*_4_ = 512, *F*_5_ = 1024, and *K*_*h*_*K*_*w*_ = *K*_*h*_ × *K*_*w*_. *K*_*h*_ = *K*_*w*_ = 5 denote the height and width of the convolution kernel, respectively. The first layer is an input layer. The layers numbered 3, 5, 8, 10, 13, 15, 18, 20, 23, 25, 29, 31, 35, 37, 41, 43, 47, and 49 are (18) activation layers. The layers numbered 6, 11, 16, and 21 are (4) max-pooling layers. The layers numbered 26, 32, 38, and 44 are (4) up-sampling layers. The layers numbered 27, 33, 39, and 45 are (4) concatenate layers. The total trainable parameters are 87,149,953.

Layer number	Layer name	Number of trainable parameters
2	Conv01	(*K*_*h*_*K*_*w*_ × *N*_*c*_ + 1) × *F*_1_ = 1664
4	Conv02	(*K*_*h*_*K*_*w*_ × *F*_1_ + 1) × *F*_1_ = 102464
7	Conv03	(*K*_*h*_*K*_*w*_ × *F*_1_ + 1) × *F*_2_ = 204928
9	Conv04	(*K*_*h*_*K*_*w*_ × *F*_2_ + 1) × *F*_2_ = 409728
12	Conv05	(*K*_*h*_*K*_*w*_ × *F*_2_ + 1) × *F*_3_ = 819456
14	Conv06	(*K*_*h*_*K*_*w*_ × *F*_3_ + 1) × *F*_3_ = 1638656
17	Conv07	(*K*_*h*_*K*_*w*_ × *F*_3_ + 1) × *F*_4_ = 3277312
19	Conv08	(*K*_*h*_*K*_*w*_ × *F*_4_ + 1) × *F*_4_ = 6554112
22	Conv09	(*K*_*h*_*K*_*w*_ × *F*_4_ + 1) × *F*_5_ = 13108224
24	Conv10	(*K*_*h*_*K*_*w*_ × *F*_5_ + 1) × *F*_5_ = 26215424
28	Conv11	(*K*_*h*_*K*_*w*_ × (*F*_5_ + *F*_4_) + 1) × *F*_4_ = 19661312
30	Conv12	(*K*_*h*_*K*_*w*_ × *F*_4_ + 1) × *F*_4_ = 6554112
34	Conv13	(*K*_*h*_*K*_*w*_ × (*F*_4_ + *F*_3_) + 1) × *F*_3_ = 4915456
36	Conv14	(*K*_*h*_*K*_*w*_ × *F*_3_ + 1) × *F*_3_ = 1638656
40	Conv15	(*K*_*h*_*K*_*w*_ × (*F*_3_ + *F*_2_) + 1) × *F*_2_ = 1228928
42	Conv16	(*K*_*h*_*K*_*w*_ × *F*_2_ + 1) × *F*_2_ = 409728
46	Conv17	(*K*_*h*_*K*_*w*_ × (*F*_2_ + *F*_1_) + 1) × *F*_1_ = 307264
48	Conv18	(*K*_*h*_*K*_*w*_ × *F*_1_ + 1) × *F*_1_ = 102464
50	Conv19	(1 × 1 × *F*_1_ + 1) × *N*_*c*_ = 65

### Loss function

For our problem, we used a mean-squared-error (MSE) training loss function, which measures the average of the squares of the errors. Given the reference solution **Y**_ref_ and the model prediction ${{\bf{Y}}}_{{\rm{p}}{\rm{r}}{\rm{e}}}$, the MSE is computed as follows:

3 $$\mathrm{MSE}=\frac{1}{L}{\mathrm{||}{{\bf{Y}}}_{\mathrm{ref}}-{{\bf{Y}}}_{\mathrm{pre}}\mathrm{||}}_{\mathrm{Fro}}^{2}=\frac{1}{L}{\mathrm{||}{{\bf{Y}}}_{\mathrm{ref}}-f\left({\bf{X}};{\boldsymbol{\theta }}\right)\mathrm{||}}_{\mathrm{Fro}}^{2},$$

where *L* is the number of elements in **Y**_ref_, ${\mathrm{||}\cdot \mathrm{||}}_{{\rm{F}}{\rm{r}}{\rm{o}}}$ being the Frobenius norm. The MSE loss function is widely used in statistics. Because we will work in a batch-wise fashion in the training process, the loss function is composed of a sum of subfunctions evaluated at different mini-batches of data. The loss function is accordingly stochastic.

### Optimization algorithm

We employed an Adam (derived from adaptive moment estimation) algorithm to optimize our stochastic loss function. Adam is a simple and computationally efficient algorithm for first-order gradient-based optimization^26^. Please see algorithm 1 for the pseudo-code of the employed Adam algorithm. *ϕ*(**θ**) denotes the stochastic scalar loss function, which is differentiable with respect to parameters *θ*. **g**_*t*_ = ▽_*θ*_*ϕ*_*t*_(**θ**) represents the gradient, i.e., the vector of partial derivatives of *ϕ*_*t*_ with respect to *θ* evaluated at time step *t*. Adam updates exponential moving averages of the gradient **m**_*t*_ and the squared gradient **v**_*t*_ where the hyper-parameters *β*_1_, *β*_2_ ∈ [0, 1) control the exponential decay rates of these moving averages. The moving averages themselves are estimates of the first moment (the mean) and the second moment (the uncentred variance) of the gradient^26^.

Algorithm 1 shows that Adam only requires first-order gradients, which is straightforward to implement with little memory requirement. Adam is designed to combine the advantages of two previously popular optimization algorithms: AdaGrad^27^, which deals with sparse gradients well, and RMSProp, which works well in on-line and non-stationary objectives^26^. Adam is based on adaptive estimates of lower-order moments. Kingma and Ba^26^ analysed the theoretical convergence properties of the Adam algorithm. Adam is a versatile algorithm for DL problems with big data and/or high-dimensional parameter spaces. Using large ANNs and data sets, Kingma and Ba^26^ found Adam to be efficient, robust and well-suited to a wide range of practical non-convex optimization problems in the DL field.

### Evaluation metrics

As mentioned above, MSE is a measure of the quality of a predictor, which is always non-negative. With our experience in the seismic data reconstruction field, we also utilized the signal-to-noise ratio (SNR) as follows:

4 $${\rm{S}}{\rm{N}}{\rm{R}}{\rm{(}}{\rm{d}}{\rm{B}})=-20{{\rm{l}}{\rm{o}}{\rm{g}}}_{10}\frac{{\mathrm{||}{{\bf{Y}}}_{{\rm{r}}{\rm{e}}{\rm{f}}}-{{\bf{Y}}}_{{\rm{p}}{\rm{r}}{\rm{e}}}\mathrm{||}}_{2}}{{\mathrm{||}{{\bf{Y}}}_{{\rm{r}}{\rm{e}}{\rm{f}}}\mathrm{||}}_{2}},$$

to assess the result’s quality. Moreover, we used two other metrics that are widely used in the computer vision super-resolution field: peak signal-to-noise ratio (PSNR) and structural similarity index method (SSIM)^28^. PSNR is computed in the following way:

5 $${\rm{P}}{\rm{S}}{\rm{N}}{\rm{R}}({\rm{d}}{\rm{B}})=10{{\rm{l}}{\rm{o}}{\rm{g}}}_{10}\left(\frac{{{\rm{M}}}^{2}}{{\rm{M}}{\rm{S}}{\rm{E}}}\right),$$

where *M* is the maximum value of elements in **Y**_ref_. The SSIM is defined as follows:

6 $${\rm{S}}{\rm{S}}{\rm{I}}{\rm{M}}\left({{\bf{Y}}}_{{\rm{r}}{\rm{e}}{\rm{f}}},{{\bf{Y}}}_{{\rm{p}}{\rm{r}}{\rm{e}}}\right)=\frac{\left(2{{\rm{\mu }}}_{{{\bf{Y}}}_{{\rm{r}}{\rm{e}}{\rm{f}}}}{{\rm{\mu }}}_{{{\bf{Y}}}_{{\rm{p}}{\rm{r}}{\rm{e}}}}+{{\rm{C}}}_{1}\right)}{\left({{\rm{\mu }}}_{{{\bf{Y}}}_{{\rm{r}}{\rm{e}}{\rm{f}}}}^{2}+{{\rm{\mu }}}_{{{\bf{Y}}}_{{\rm{p}}{\rm{r}}{\rm{e}}}}^{2}+{{\rm{C}}}_{1}\right)}\times \frac{\left(2{{\rm{\sigma }}}_{{{\bf{Y}}}_{{\rm{r}}{\rm{e}}{\rm{f}}}{{\bf{Y}}}_{{\rm{p}}{\rm{r}}{\rm{e}}}}+{{\rm{C}}}_{2}\right)}{\left({{\rm{\sigma }}}_{{{\bf{Y}}}_{{\rm{r}}{\rm{e}}{\rm{f}}}}^{2}+{{\rm{\sigma }}}_{{{\bf{Y}}}_{{\rm{p}}{\rm{r}}{\rm{e}}}}^{2}+{{\rm{C}}}_{2}\right)},$$

where ${{\rm{\mu }}}_{{{\bf{Y}}}_{{\rm{r}}{\rm{e}}{\rm{f}}}}$ and ${{\rm{\mu }}}_{{{\bf{Y}}}_{{\rm{p}}{\rm{r}}{\rm{e}}}}$ denote the mean of **Y**_ref_ and ${{\bf{Y}}}_{{\rm{p}}{\rm{r}}{\rm{e}}}$, respectively; ${{\rm{\sigma }}}_{{{\bf{Y}}}_{{\rm{r}}{\rm{e}}{\rm{f}}}}$ and ${{\rm{\sigma }}}_{{{\bf{Y}}}_{{\rm{p}}{\rm{r}}{\rm{e}}}}$ represent the variance of **Y**_ref_ and ${{\bf{Y}}}_{{\rm{p}}{\rm{r}}{\rm{e}}}$, respectively; ${{\rm{\sigma }}}_{{{\bf{Y}}}_{{\rm{r}}{\rm{e}}{\rm{f}}}{{\bf{Y}}}_{{\rm{p}}{\rm{r}}{\rm{e}}}}$ is covariance between **Y**_ref_ and ${{\bf{Y}}}_{{\rm{p}}{\rm{r}}{\rm{e}}}$. The constant *C*_1_ is included to avoid instability when ${{\rm{\mu }}}_{{{\bf{Y}}}_{{\rm{r}}{\rm{e}}{\rm{f}}}}^{2}+{{\rm{\mu }}}_{{{\bf{Y}}}_{{\rm{p}}{\rm{r}}{\rm{e}}}}^{2}$ is very close to zero. Similarly, the constant *C*_2_ is developed to avoid instability if ${{\rm{\sigma }}}_{{{\bf{Y}}}_{{\rm{r}}{\rm{e}}{\rm{f}}}}^{2}+{{\rm{\sigma }}}_{{{\bf{Y}}}_{{\rm{p}}{\rm{r}}{\rm{e}}}}^{2}$ is very close to zero.

**Algorithm 1:**
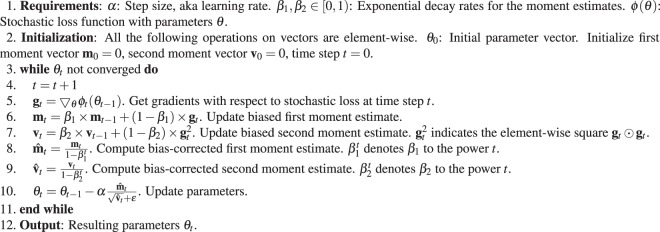
Adam, employed optimization algorithm for stochastic loss function.

The metrics MSE, SNR, PSNR, and SSIM can be used in the training and test processes to quantify the performance of the result. The MSE values closer to zero are better. Generally, the higher the SNR, PSNR, and SSIM values are, the better the result.

### Training preparation and setup

#### Training data

The training data are vital for DL. We prepared the training data utilizing not only the synthetic data but also the field seismic data, to let the model learn the features of seismic data by being given as many instances as possible.

**Figure 3:**
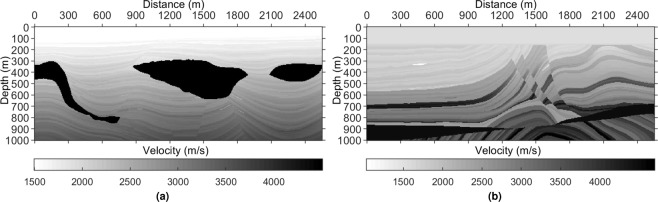
Velocity models used to generate the synthetic data. Each velocity model has its own density model. (**a**) Adapted Pluto 1.5 model. (**b**) Down-sampled Marmousi2 model. The lateral spacing dx and the depth spacing dz are 5 m.

**Figure 4:**
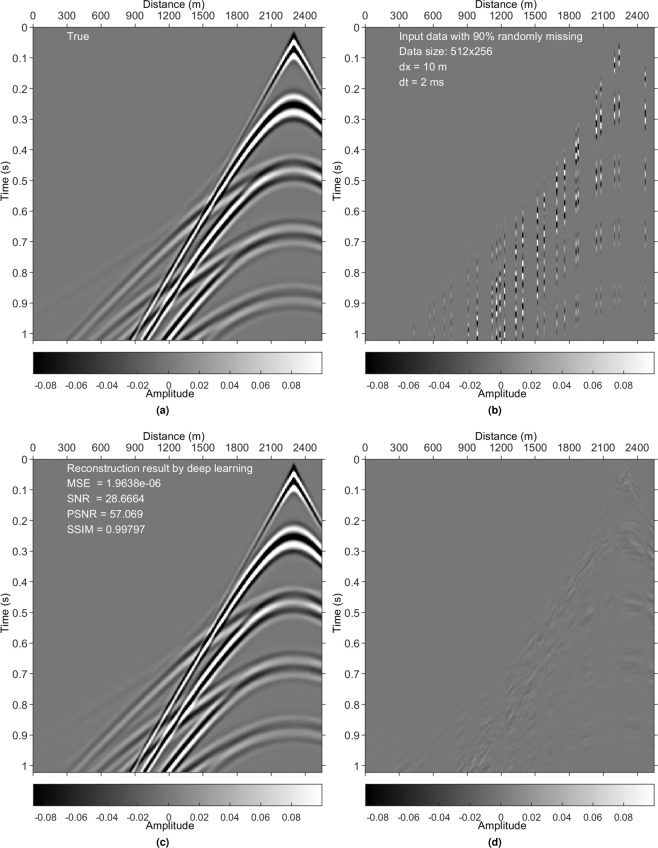
Results on a synthetic training data set. (**a**) True data (a common-shot-point, CSP, gather). (**b**) Irregularly sampled data with 90% missing. (**c**) DL reconstruction result. (**d**) Difference between (**a**,**c**).

The synthetic training data are modelled with a forward-modelling code^29^ based on some well-designed earth models (e.g., Fig. 3a). The model in Fig. 3b is used to generate the test data that are completely unseen in the training process. The sources and receivers are equally distributed from 0 to 2550 m with 10 m spacing. The source is shifted from the location of the first shot to the last one. The source and receiver depths are changed for different earth models and experiments. The simulated data include 2048 shots (8 simulations, 256 shots per simulation), and 256 traces per shot. There are 2048 samples along the time axis with a time interval dt of 0.5 ms. Because dt = 0.5 ms is barely used in industry, we revised the data of size 2048 × 256 × 2048 (arranged in the order of time axis, receiver axis, and shot axis) to three data sets: 1024 × 256 × 2048 (dt = 1 ms), 512 × 256 × 2048 (dt = 2 ms), and 256 × 256 × 2048 (dt = 4 ms), composing the synthetic training data. Figure 4 shows a sample.

**Figure 5:**
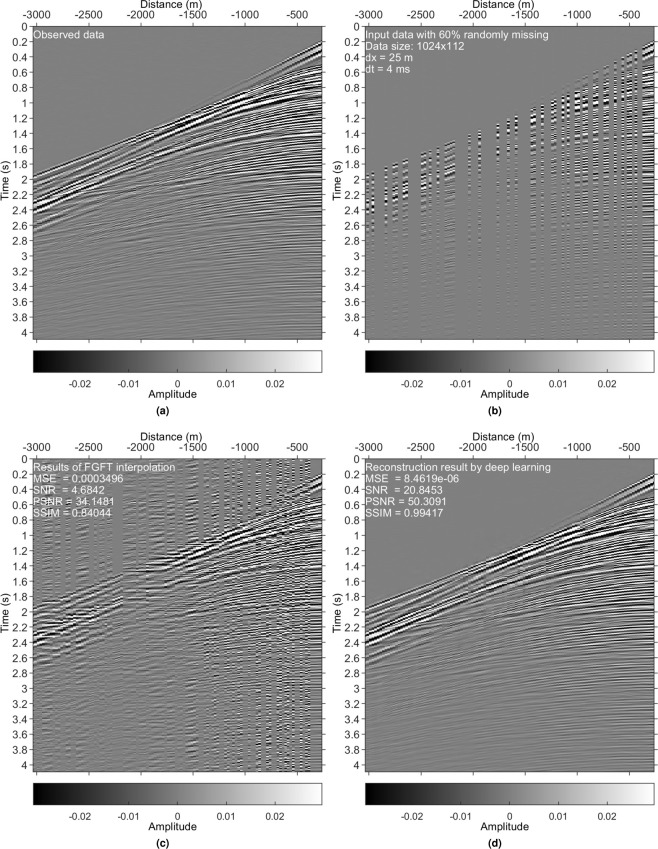
Results on the Mobil Viking graben line 12 data set. (**a**) Reference data. (**b**) Irregularly sampled data with 60% missing. (**c**) Interpolated data using the FGFT interpolation method^23^. (**d**) DL reconstruction result.

We exploited the Mobil Viking graben line 12 data set to generate the field training data. This data set is composed of 1001 shot gathers. Each gather is of size 1024 × 120: 1024 rows represent the time domain, sampled every 4 ms; 120 columns are in the spatial domain with 25 m of sampling. We randomly choose 200 shots as the field training data set (see Fig. 5 for a sample).

Before being fed into the model, each shot gather is normalized by dividing the maximum value of the absolute value of the corresponding shot gathers. Consequently, the amplitudes of the model input and output are finally in the range [−1, 1]. To ensure a sufficiently large number of data samples for learning, we work in a patch-wise fashion. The shot gathers are divided into small patches with a specified size. The training data in terms of patches is much larger than the number of training shot gathers.

#### Training setup

There is a trade-off between the patch size (determining the receptive field) and the model depth. A larger patch size demands more down- and up-sampling layers, while small patches allow the model to see only local features. In addition, we should select the patch size such that all 2 × 2 down-sampling operations can be applied to a layer with an even height and width size. The patch size and the batch size are primarily limited by the graphics processing unit (GPU) memory. To minimize the overhead and make full use of the GPU memory, we prefer a large patch size over a large batch size.

**Figure 6:**
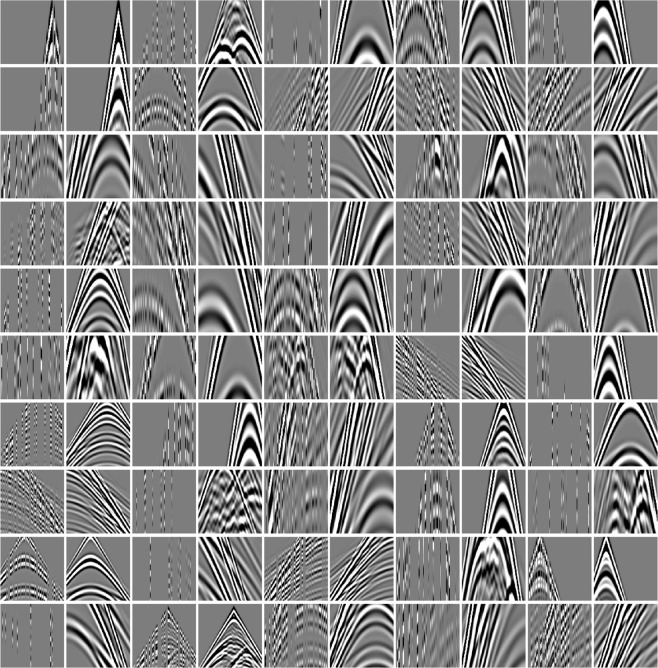
Model input-output pairs, which are randomly selected from the training data set. Each pair is composed of irregularly missing incomplete data (in the odd column, i.e., the model input **X**) and the corresponding complete data (in the even column, i.e., the model output **Y**_ref_). The size of each panel is 112 × 112.

Our available computing resources are summarized as follows: a workstation with Windows 7, two Intel Xeon E5-2620 processors, 2.10 GHz CPU, 176 GB of RAM, and an NVIDIA GeForce RTX 2080 Ti GPU (11 GB). Our codes are written in Python based on Keras (a Python DL library). After trial and error, the patch size is set as 112 × 112, which allows four times of down-sampling operations for the field training shot gather with 120 traces. To overlap adjacent patches, the patch-stride is 23 pixels for the synthetic training data sets, and 10 pixels for the field training data set. Patches with a smaller mean absolute value (e.g., ≤0.001) indicate that there are few events located within the patch or these amplitude values are nearly zeros. The patches below a threshold value are removed from the training data. Then, we paid special attention to the first-arrival areas as their samples are fewer in comparison to other areas. As a result, we augment the proportion of the samples belonging to the shallow first-arrival areas to some degree. The training process finally involves 1,132,800 (more than 1 million) patches. We set the batch size as 128. The steps-per-epoch is set as 8850, which denotes the total number of steps (batches of samples) before declaring one epoch finished and starting the next epoch. The steps-per-epoch is typically equal to the number of samples of the training data set (1,132,800) divided by the batch size (128). Figure 6 demonstrates fifty model input-output training pairs.

The speed provided by computation of the updates on small batches of data, in parallel, on specialized hardware (e.g., GPU) allows one to fit networks with millions of parameters on data sets with millions of observations. Good default settings of Adam for the tested DL problems are *β*_1_ = 0.9, *β*_2_ = 0.999, and *ε* = 10^−8^. The learning rate *α* is initialized at 0.0001, which is a critical parameter. Nevertheless, determining how to obtain optimal values of the learning rate is still an open issue. The number of epochs should be specified to train the model. Too few epochs generate a poor under-fitting DL result, and too many epochs waste running time and possibly produce over-fitting results. With our experience, 50 epochs obtain sufficiently good results. Moreover, the indexes of the input-output pairs are shuffled before starting the next epoch. In the end, to ensure the original available data remain unaltered, the live data from the original input are reinserted into their original positions in the DL result.

## Results

**Figure 7:**
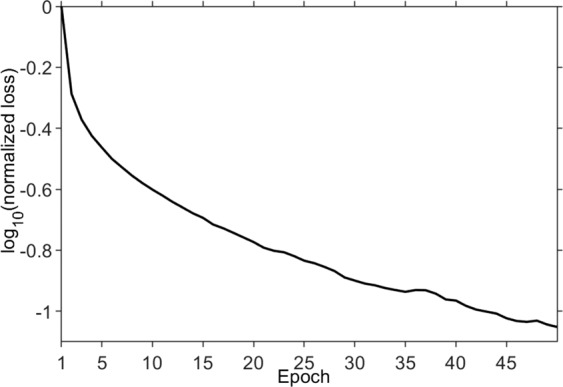
During training, variation of ${{\rm{l}}{\rm{o}}{\rm{g}}}_{10}({\rm{n}}{\rm{o}}{\rm{r}}{\rm{m}}{\rm{a}}{\rm{l}}{\rm{i}}{\rm{z}}{\rm{e}}{\rm{d}}\,{\rm{l}}{\rm{o}}{\rm{s}}{\rm{s}})$ with epoch.

**Figure 8:**
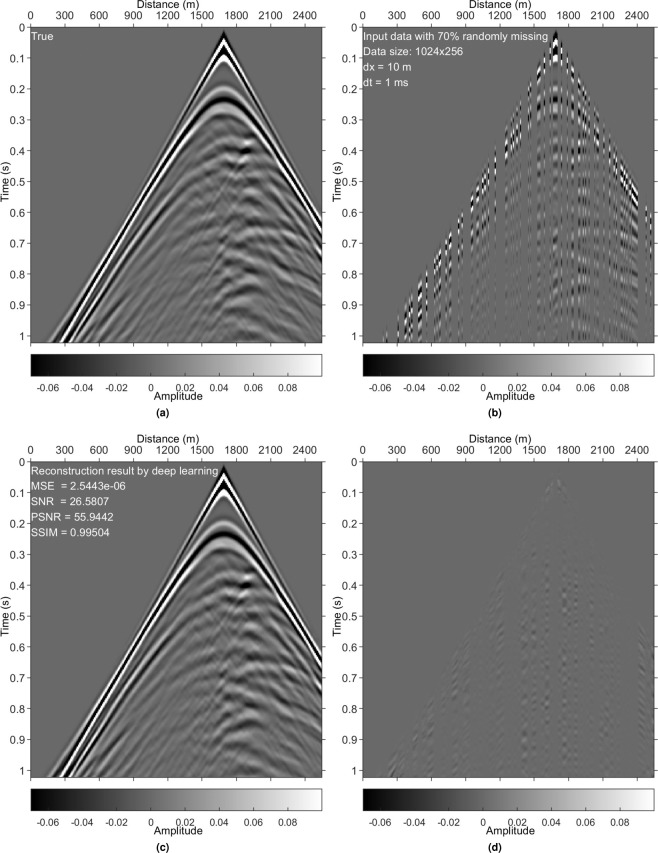
Results on a test data set generated with the Marmousi2 model. (**a**) True data. (**b**) Irregularly sampled data with 70% missing. (**c**) DL reconstruction result. (**d**) Difference between (**a**,**c**).

**Figure 9:**
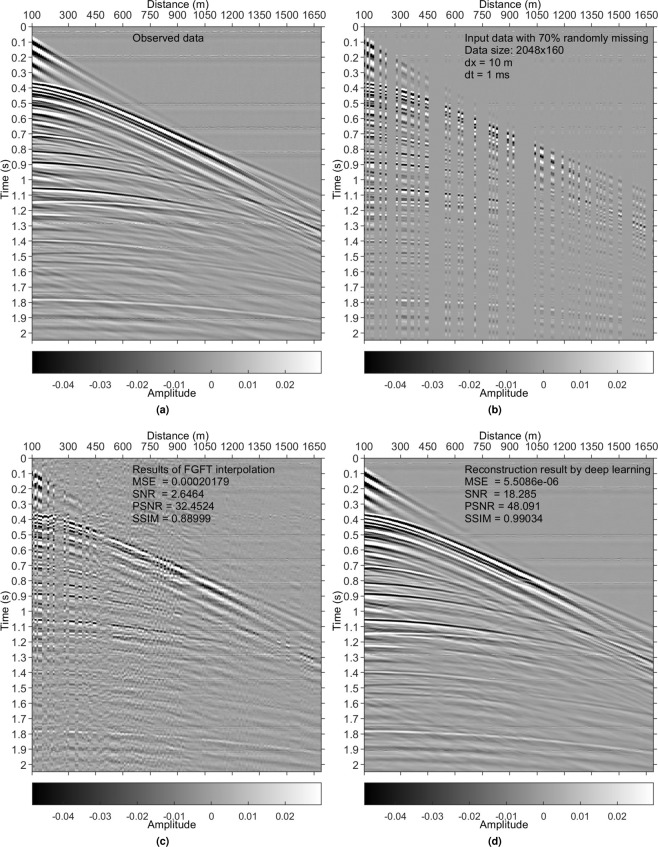
Results on a physical modelling data set^30^. (**a**) Reference data. (**b**) Irregularly sampled data with 70% missing. (**c**) Interpolated data using a fast-generalized Fourier transform (FGFT) method^23^. (**d**) DL reconstruction result.

**Figure 10:**
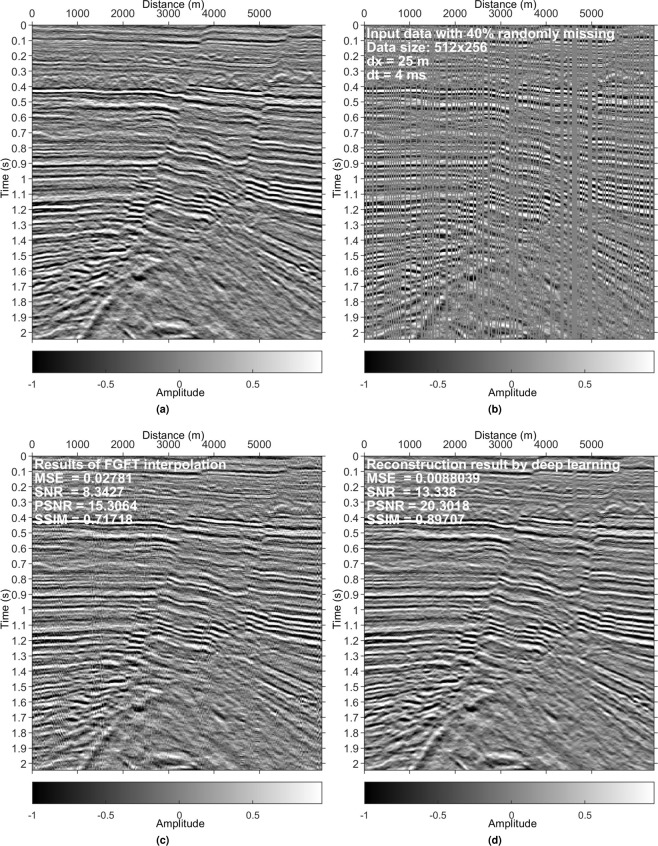
Results on a data set from the GeoFrame software. (**a**) Reference data. (**b**) Irregularly sampled data with 40% missing. (**c**) Interpolated data using the FGFT interpolation method^23^. (**d**) DL reconstruction result.

**Figure 11:**
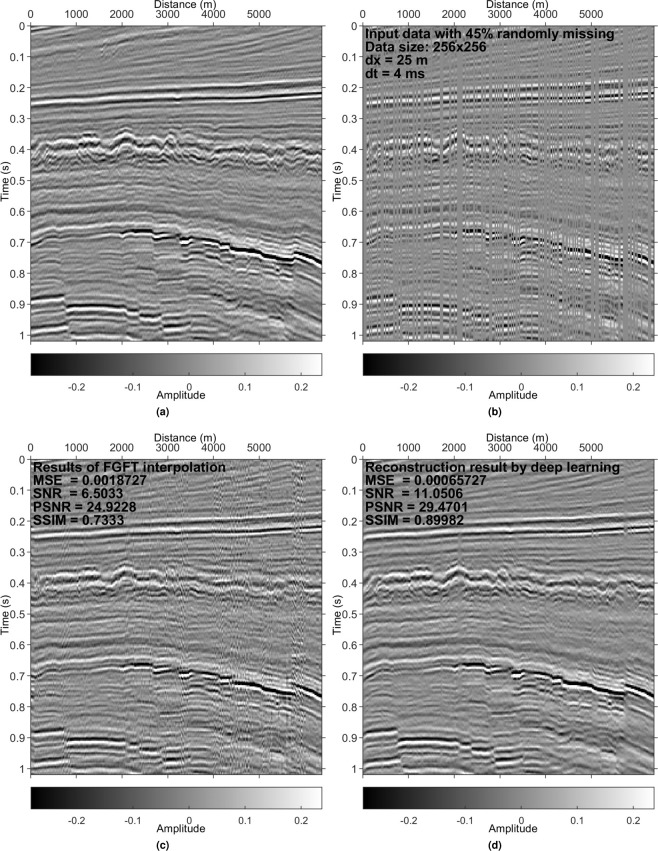
Results on the F3 data set. (**a**) Reference data. (**b**) Irregularly sampled data with 45% missing. (**c**) Interpolated data using the FGFT interpolation method^23^. (**d**) DL reconstruction result.

**Figure 12:**
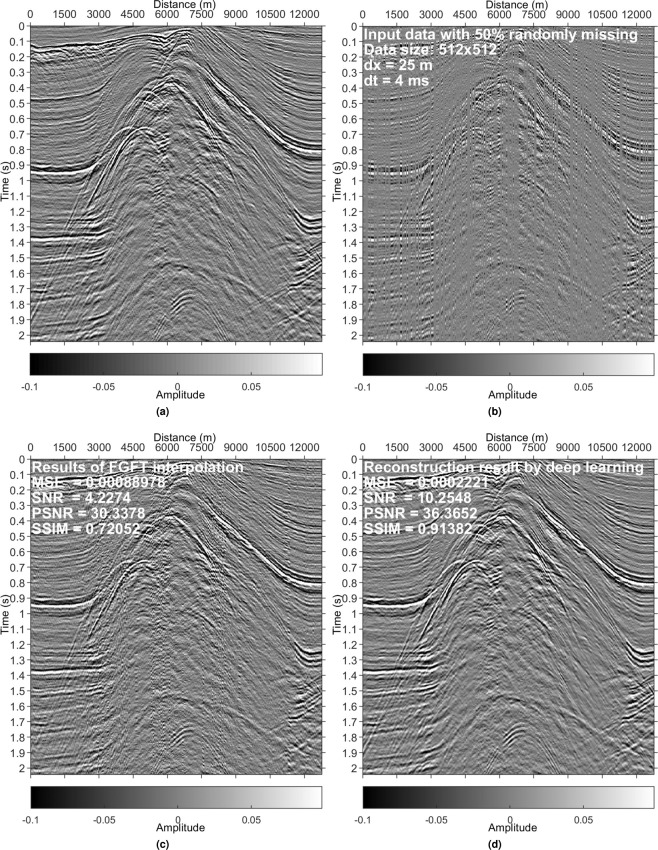
Results on a data set from the North Sea. (**a**) Reference data. (**b**) Irregularly sampled data with 50% missing. (**c**) Interpolated data using the FGFT interpolation method^23^. (**d**) DL reconstruction result.

**Figure 13:**
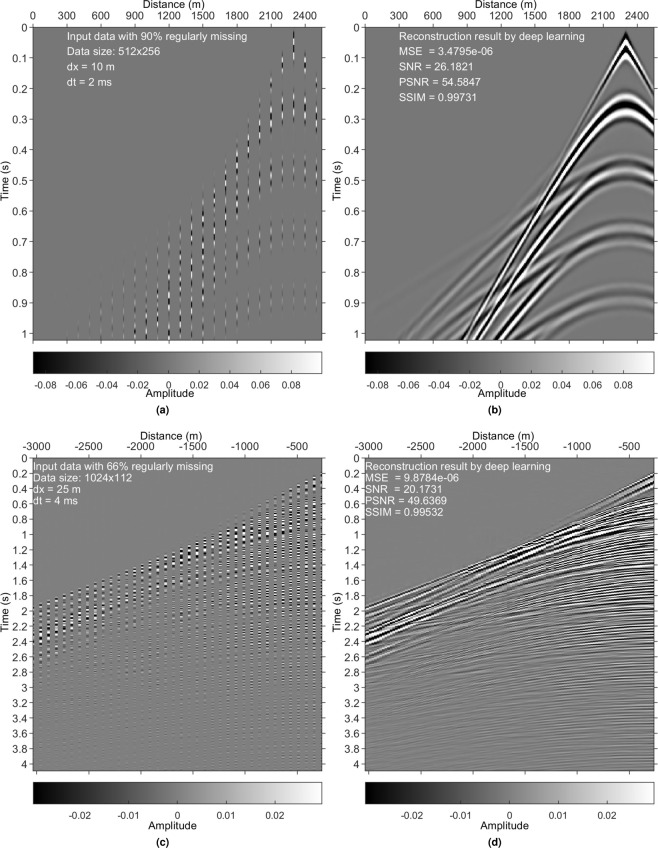
Regularly missing data reconstruction. (**a**) Regularly sampled data from the synthetic training data set with a decimation factor of 10 in the space direction (90% missing). (**b**) DL result for (**a**). (**c**) Regularly sampled data from the Mobil Viking graben line 12 data set with a decimation factor of 3 in the space direction (66% missing). (**d**) DL result for (**c**).

In the training process, the model input is restricted in a specified patch size (112 × 112). However, for the model test, the input data need not to be divided into small patches. That is, a profile can be directly fed into the model. One of the most important issues is convergence of the training process. The training log shown in Fig. 7 indicates convergence, which is successively going down with the increasing epoch numbers. For a well-trained model, it should produce reasonable output for new input that is never seen in the training process (aka the model’s generalization capability). We exploit several typical data sets (e.g., those shown in Figures 5, 8–13) to test the generalization capacity. Because we work in a local patch-wise fashion in the training process, the complete feature of a shot gather belonging to the training data (e.g., Fig. 4) is not completely seen. Application of the trained model to irregularly missing data reconstruction is first on the agenda; then, regularly missing data reconstruction follows.

### Irregularly missing data reconstruction

Examples in Figs. 4, 5, 8 and 9 validate that the trained model can reconstruct irregularly missing data with high accuracy. The model output, seen in Figs. 4 and 8, shows negligible difference between the true data and the DL result. To check the superiority of the new method over conventional methods, we compare it with a peer-reviewed FGFT interpolation method^23^. The code of the FGFT method is open-source. We made no change to the code of the FGFT method. There are few adjustable parameters of the FGFT method. The results of the FGFT method are correspondingly shown in Figs. 5, 9–12. Though the parameter may be not the optimal one, Figs. 5, 9–12 can show the drawbacks of the FGFT method to some degree. By comparing the FGFT interpolation results with those of DL, DL achieves smaller MSE values and higher SNR, PSNR, SSIM values. These results verify the feasibility, effectiveness, superiority, and generalization capacity of the evaluated method.

Comparing Figs. 4, 5, 8 and 9 (pre-stack data applications) with Figs. 10–12 (post-stack data applications) reveals that reduced precision emerges (reflected in the relatively lower SNR values in Figs. 10–12) if the features of the test data are significantly different from those of the training data. Note that the model is trained with pre-stack data only. The bias increases as the differences increase. Even though the performance decreases with the increasing feature difference between training and test data, the model still generates acceptable results in comparison to the FGFT results (see Figs. 10–12). We think different types of "reliable” data should be added to the training data to further improve the model’s generalization capability.

### Regularly missing data reconstruction

Our primitive goal is to accomplish the irregularly missing data reconstruction. We did not realize that the trained model is suitable for the regularly missing case. Excited by successful tests (two of them are shown in Fig. 13), we found that the evaluated framework is also competent in regularly missing data reconstruction. The reason is that regularly missing can be seen as a special case of irregularly missing. The model trained with irregularly sampled data can be applied to regularly missing data reconstruction. However, the model trained with regularly sampled data cannot be applied to irregularly missing data reconstruction.

## Discussion

DL is a promising data-driven approach for solving inverse problems and, by extension, data reconstruction tasks. The model as established in this work may have tens to hundreds of millions of trainable parameters (see Table 1, approximately 87 million), giving rise to a large GPU memory requirement. The key computational cost of DL rests in the training process. However, it occurs once up front. The computational cost of model prediction is inexpensive. For example, the prediction of a 1024 × 112 shot gather costs less than 2 s on a computer without using the GPU. Hence, the overall computational cost is efficient.

Although we have concentrated on 2D, our method can be generalized to 3D/5D cases. A generalization to 3D demands substituting the 2D convolution/pooling/up-sampling layers with 3D versions, which is supported by numerous DL frameworks (e.g., Keras, TensorFlow, and PyTorch). We are moving towards 3D/5D reconstruction with the hope of obtaining superior results by using more spatial constraints. In this work, we have focused on missing data reconstruction, but the framework presented here also suggests similar potentials of DL in other fields (e.g., super-resolution reconstruction of photos and maps, signal processing, and imaging). Once a general model architecture is ready, the same idea can be applied to many problems.

## Conclusions

We assessed a deep-learning-based framework for both irregularly and regularly missing data reconstruction, which is aimed at transforming incomplete data into their corresponding complete data. For achieving this goal, we first build a network architecture with the randomly sampled incomplete data as the model input and the corresponding complete data as the model output, which is based on an encoder-decoder-style end-to-end U-Net CNN. Then, we use a mean-squared-error loss function and an Adam optimization algorithm to train the model. Next, we prepare the training data utilizing both synthetic and field seismic data. We describe the established model architecture, the used loss function, the employed Adam optimization algorithm, the training data and the training setups in detail. We demonstrate the feature maps for a randomly sampled data set going through the trained model, with the aim of trying to explain how the missing data are reconstructed. We test the trained model with several typical data sets for irregularly missing data reconstruction, which achieves better performances compared with the FGFT interpolation method, verifying the feasibility, effectiveness, superiority, and generalization capability of the evaluated framework. Because regularly missing data can be considered as one special case of irregularly missing data, the trained model is also successfully applied to regularly missing data reconstruction. This work supports that DL can avoid some assumptions limiting conventional interpolation methods (e.g., assumptions of linear events, sparseness, and low-rank) and possesses great potential in advanced intelligent applications over traditional techniques.
